# Clinical utility of polygenic risk scores in cardiovascular disorders

**DOI:** 10.1515/medgen-2026-3008

**Published:** 2026-07-08

**Authors:** Heribert Schunkert, Ling Li, Teresa Trenkwalder

**Affiliations:** TUM University Hospital Department of Cardiology Lazarettstr. 36 80636 Munich Germany; TUM University Hospital Department of Cardiology Lazarettstr. 36 80636 Munich Germany; TUM University Hospital Department of Cardiology Lazarettstr. 36 80636 Munich Germany

**Keywords:** polygenic risk score, coronary artery disease, atrial fibrillation, heart failure, cardiomyopathy

## Abstract

The sudden and often fatal onset of many cardiovascular disease (CVD) calls for improved risk prediction and personalized preventive strategies. Polygenic risk score (PRS) captures inherited susceptibility and may add precision to guideline-recommended clinical risk score. Given the spectrum of CVD, the utility of PRS varies from being a clinically meaningful diagnostic tool to a scientific instrument that brings light into the complex aetiologies. This review summarizes the current understanding of PRS in CVD. We discuss methodological strategies for its construction and outline clinical scenarios for PRS across major CVDs, including coronary artery disease, aortic aneurysms, cardiomyopathies, atrial fibrillation in the context of heart failure, and hypertension, highlighting their potential to enhance risk stratification beyond traditional factors. Finally, we outline current knowledge gaps that limit the clinical implementation of PRS.

## Introduction

The manifestation of cardiovascular disease (CVD) is influenced by a wide spectrum of genetic effects [1]. Familial hypercholesterolemia, cardiomyopathies, or inherited arrhythmia syndromes can be caused by pathogenic variants that follow Mendelian inheritance patterns. However, they account for only a small fraction of the large burden of CVD in our population. In contrast, common cardiovascular conditions, including coronary artery disease (CAD), stroke, atrial fibrillation (AF), heart failure (HF), and valvular heart diseases, typically exhibit a polygenic architecture. This is even more pronounced for common CVD risk factors like hypertension, lipid disorders, or diabetes mellitus.

**Figure 1:**
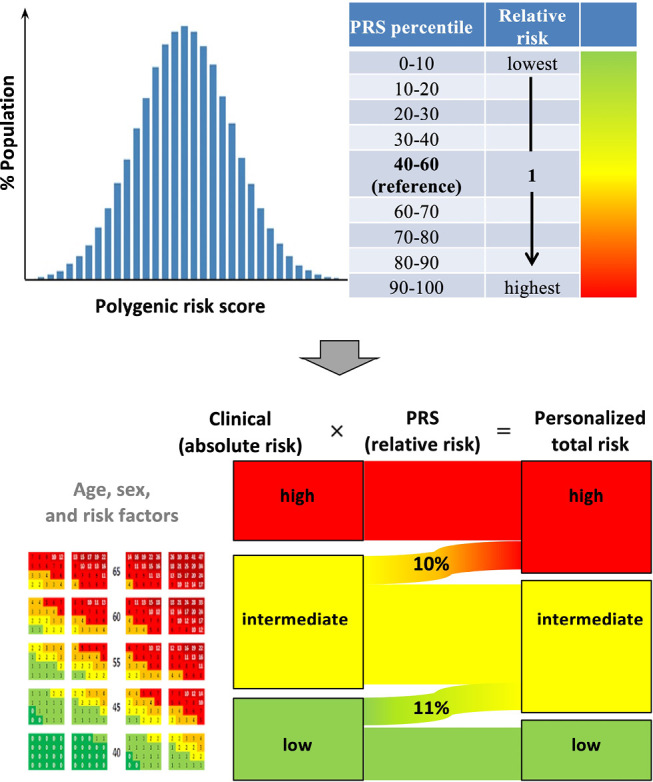
** Clinical Application of Polygenic Risk Score.** Risk prediction for CVD typically starts with a clinical risk assessment. For coronary artery disease, this includes risk factors such as hypertension, smoking or diabetes mellitus. Clinical risk scores estimate **absolute** event rates over time (e.g., 5 % within 10 years). Data from discovery GWAS allow to build a polygenic risk score (PRS), which stratifies individuals based on their **relative** genetic risk. Compared to the average, this may range from low to high, usually a factor from 0.4 to 4. Combining the clinical estimate with the relative PRS risk offers a personalized total risk estimate which improves risk classification accuracy. As illustrated in the figure, a UK Biobank-based study suggests that incorporating PRS into SCORE2 reclassifies approximately 11 % of individuals from low to intermediate risk and about 10 % from intermediate to high risk[16]. Examples are given in Figure 2.

Within the last two decades, genome-wide association studies (GWAS) have identified hundreds of loci that contribute to these common CVDs [2–4]. These findings indicated that the high SNP-based heritability (*h*^2^_SNP_) of common CVD, e.g., 20–40 % for CAD [2, 5] or 20–50 % for blood pressure [6], is best explained by the cumulative action of many common variants with weak effects. Aside from giving empirical proof to the *common-disease – common-variant* hypothesis, these discoveries led to novel biological concepts, drug developments as well as improved clinical risk prediction.

Currently, prediction models for most CVDs rely on age and conventional risk factors, except for certain cardiomyopathies [7] where monogenic high-risk variants are considered as main underlying causes [8]. Although common genetic variants contribute substantially to CVD susceptibility, polygenic risk scores (PRS) have not been incorporated into guideline-based risk assessment yet. However, this may change with improving accuracy and broader applicability of PRS [9]. Indeed, PRS offers a promising approach for earlier and more personalised cardiovascular risk prediction and therapeutic precision (Figure 1). Importantly, polygenic factors can also act as genetic modifiers in monogenic disorders, contributing to variability in penetrance and phenotype [10].

## Interpretation of PRS

A PRS is typically calculated as a weighted sum of risk alleles derived from discovery GWAS [11]. It generally follows a Gaussian distribution and measures relative risk compared with a reference population [12]. For example, the CAD prevalence of individuals in the top decile of a CAD-PRS is twice as high as that of the population average and those in the top 0.5 % carry an even five-fold increased risk [13, 14].

The PRS contrasts with the clinical scores, which incorporate age, sex, and traditional clinical factors to provide estimates of absolute risk over a 10-years period. For example, young individuals usually have a low CAD risk according to SCORE2, the score recommended by the European Society of Cardiology (ESC), in which age is the most important risk indicator [7]. In this context, a PRS may be a useful risk-stratification tool since young individuals may be rather interested in their lifetime risk, which may vary substantially depending on their polygenic risk [14, 15]. In middle-aged people, the SCORE2 may offer relevant clinical information, because the absolute risk estimate from the clinical score can be multiplied by the relative risk (e.g. odds ratio) of the PRS [16].

In addition, PRS performance is ancestry-dependent because GWAS effect sizes vary across populations due to differences in linkage disequilibrium (LD), allele frequencies, or ancestry-specific risk [2]. Since most GWAS are based on European samples, PRS often show reduced accuracy in other ethnic groups, motivating the development of multi-ancestry PRS to improve cross-population prediction [17, 18].

## Methodological aspects for clinical use of PRS

Building a PRS involves three main stages: quality control, PRS calculation, and performance evaluation. First, both discovery GWAS data and target genotype data need to undergo stringent quality control [19]. The PRS is then derived using either clumping and thresholding (e.g., PLINK[20] and PRSice-2 [21]) or more sophisticated models such as LDpred-2 (Bayesian) [22] and lassosum (penalized regression) [23], which enhance accuracy by modelling linkage disequilibrium. New approaches, like machine learning and tools integrating multi-ancestry panels (e.g., PRS-CSx), further enhance portability and predictive power [24]. The performance of PRS is measured using metrics such as area under the curve (AUC), effect size (odds ratio [OR] or hazard ratio [HR]), or variance explained (R²).

### Strategy of building PRS

A comparison of three strategies for constructing PRS is provided in Table 1.

A *single-trait PRS* (stPRS) is calculated as the weighted sum of risk alleles:


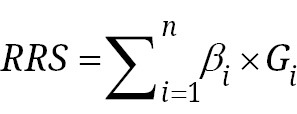


where 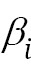 is the effect size of variant *i*, 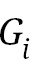 is the variant’s genotype dosage, and *n* is the number of variants. Due to its simplicity, this framework remains the standard in genetic epidemiology.

*Multi-trait PRS* (mtPRS) leverages genetic correlations between related phenotypes to improve effect size estimation [25]. It can be constructed by joint modelling of multiple PRS into one model:


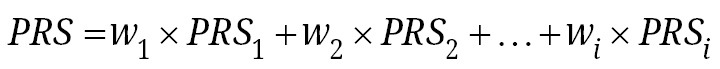


where 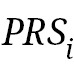 is stPRS for trait *i*, and 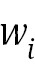 is its weight. Integration methods range from simple approaches such as combinations using principal component analysis to sophisticated machine learning frameworks [26].

Alternatively, mtPRS constructs a single PRS using GWAS summary statistics leveraging cross-trait covariance. Methods such as MTAG (Multi-Trait Analysis of GWAS) [27] jointly analyse genetically correlated traits to produce refined SNP effect sizes for PRS construction. CVD illustrates this approach well as it shares substantial genetic architecture with risk factors such as lipid levels, blood pressure, type 2 diabetes, body mass index, or smoking behaviours [28]. Because CAD PRS is also predictive for stroke or peripheral arterial disease risk, integrating these traits may improve prediction and better align with clinical risk scores.

**Table 1:** Strategy of constructing PRS

Feature	Single-trait PRS	Multi-trait PRS	Integrated clinical PRS
**Definition**	Score built on genetic data for a** single trait**.	Score built on genetic data for **multiple related traits**.	Score that combines **PRS** with **clinical risk.**
**Core Logic**	Only genomic information is needed.	Leverages **shared genetic architecture** of a multifactorial disease.	Combines **absolute clinical risk** with **relative polygenic risk**.
**Data Input**	GWAS summary data for the target disease only.	PRS for the target disease and correlated traits (e.g., risk factors to better predict CVD)	GWAS summary data (PRS) + a clinical risk score.
**Data Output**	**Genetic risk**. Relative to population average.	**Genetic risk**. Relative to population average.	**Total risk**. Expected event rate (percent per time interval).

In practice, PRS model is most valuable when integrated with clinical risk to estimate the total risk a person carries. Within such model, PRS acts as a risk-enhancing factor that can reclassify individuals into higher-risk categories where preventive interventions may be beneficial. Therefore, integrated model combining genetic predisposition with clinical variables, an* integrated clinical PRS*, can effectively guide personalized decision-making (Table 1).

## Applications in major cardiovascular disorders

### Coronary artery disease

Coronary artery disease (CAD) represents the most extensively studied application for PRS in cardiovascular medicine. Large-scale GWAS have identified more than 300 genome-wide significant risk loci for CAD [1], enabling the development of highly predictive CAD-PRS that identify individuals at markedly elevated lifetime risk [29].

Aragam et al. constructed a stPRS model incorporating over two million SNPs and achieved a HR per SD of 1.56 [30]. Subsequently, the group constructed a mtPRS model, CHDBioPRS, by integrating a CAD-specific PRS model with a biomarker-based PRS model derived from 10 CAD-related biomarkers [31]. This integrative approach further improved predictive performance HR per SD to 1.90.

The relative risk conferred by PRS is independent of traditional risk factors, thereby providing complementary information [14]. This property enables the PRS odds ratio to function as a simple multiplicative factor alongside clinical risk estimates in calculating an individual’s overall CAD risk [16].

**Figure 2:**
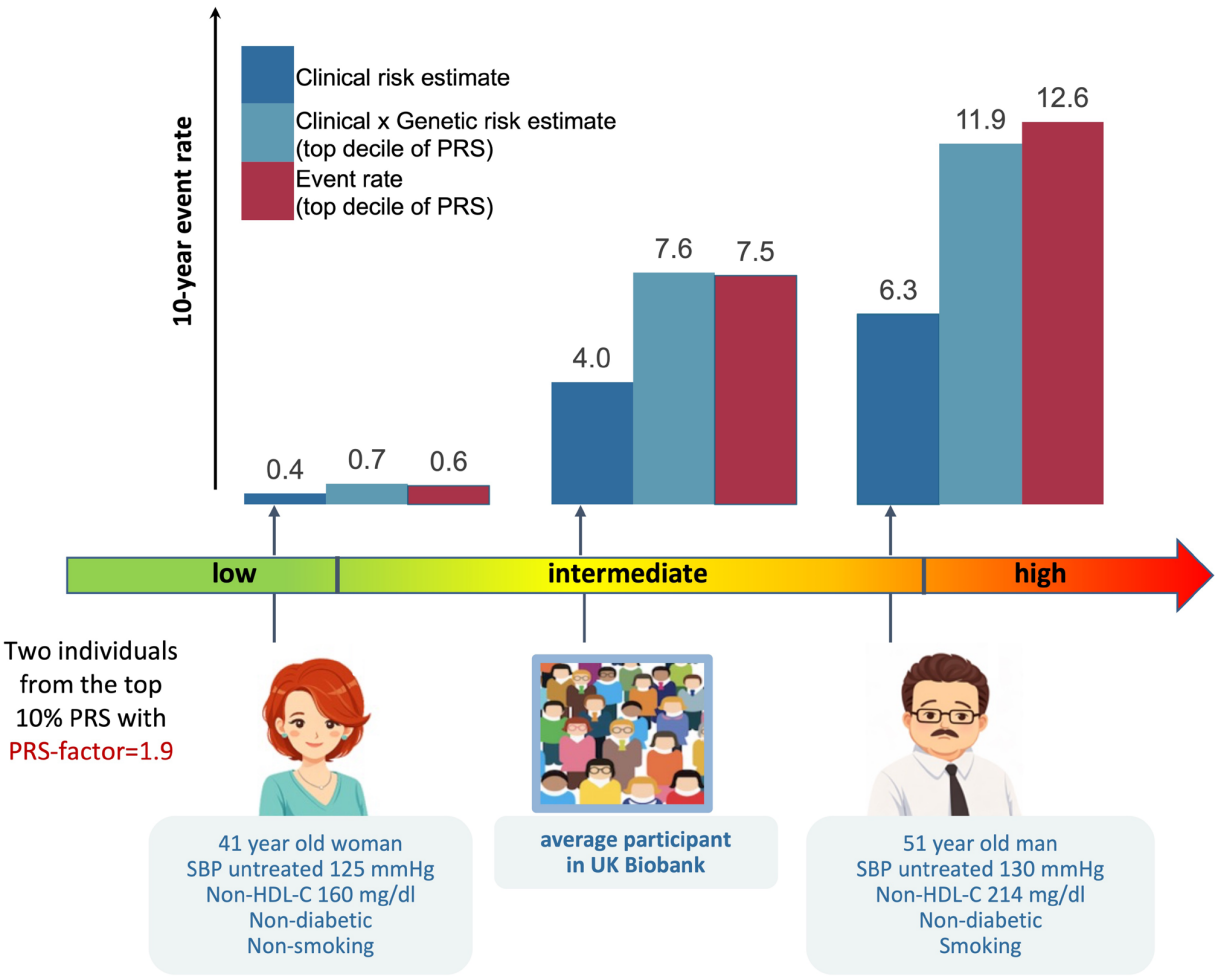
** Risk reclassification by an integrated model.** The guideline-recommended tool, SCORE2, calculates 10-year event rates based on traditional risk factors (steel blue bars; left). The integrated model (SCORE2 × PRS-factor)[16] shows the estimated event rates for respective individuals in the top decile of the CAD PRS distribution, i.e. with a PRS-factor of 1.9 (cadet blue bars, middle). The event rates found for such individuals in the UK Biobank are shown in dark red bars (right). Incorporating genetic risk into clinical risk assessment results in reclassification of some individuals from lower risk (steel blue bars; based on SCORE2 alone) to higher risk categories (cadet blue bars, integrated model), with estimated risks more closely aligned with observed incidence (dark red bars). The reclassification is particularly informative for cases like the 51-year-old man, whose clinical score hovers near the treatment threshold; the PRS factor moves him into a higher risk stratum, warranting early intervention.

Established clinical risk frameworks, such as SCORE2, PCE, and QRISK3, are based primarily on traditional risk factors[16,32,33]. An integrated model incorporating both components – for example, *SCORE2 × PRS-factor* [16] – could make genetic information accessible for clinical risk use, since the implications of a SCORE2 result are already elaborated in great detail in the guidelines. Such an approach may thus enhance risk stratification and improve clinical utility, particularly for individuals with a clinical risk estimate that is ambiguous with respect to medical treatment (Figure 1–2). The integrated clinical PRS model reclassified about 10 % of individuals initially classified as intermediate-risk group by SCORE2 alone into a higher total risk group (Figure 1). Notably, the subgroups upgraded by PRS from intermediate to high risk exhibited an incident event rate comparable to that of the original high-risk group [16]. The reclassification substantially increased the number of individuals correctly identified as being at high risk, underscoring the clinical significance of an integrated model.

A clinical consensus statement on the clinical utility of CAD-PRS was issued by a number of working groups from the ESC emphasizing the potential of PRS in refining the risk stratification beyond traditional risk scores [34]. Two clinical scenarios appeared to be of particular relevance for the clinical application of PRS.

First, in a young person who is interested in lifetime risk. At young age, clinical risk scores cannot be applied as they usually start prediction above the age of 40 years, and do so only for the period of 10 years. Moreover, typical risk factors are rarely found at young age. Knowing that the average lifetime CAD risk for a man is about 12 %, the PRS, which remains unchanged throughout life, may diversify the estimate for this person between 5–40 %.

The second scenario applies to a person with an intermediate clinical risk asking whether preventive medication is indicated. Again, given that the clinical risk estimate may vary by a factor of four, depending on the PRS, it may be sensible to consider this information before decision-making. Specifically, if the 10-year risk is 5 %, it may very well be 2.5 or 10 %, depending on the PRS, which has obvious implications for the use of diagnostic tools or preventive medication. An example was given in Figure 2, incorporating relative genetic risk (PRS-factor = 1.9) to clinical risk score reclassifies the 51-year-old man from intermediate risk to high-risk category.

Beyond polygenic risk captured by CAD PRS, cardiovascular risk can also arise from strong inherited factors with largely monogenic regulation. Lipoprotein(a) [Lp(a)] is predominantly a monogenic cardiovascular risk determinant. Approximately 70 – 90 % of interindividual variation is heritable, with the *LPA* gene accounting for > 90 % of the variance in circulation [35, 36]. Indeed, this locus was the second to show genome-wide significant association with CAD [37]. The major genetic determinants associated with elevated Lp(a) levels are variation in the high repeats of KIV-2 region in the *LPA* gene and common variants, such as rs10455872 and rs3798220 [35, 38 – 41]. Two PRS strategies have emerged for assessing cardiovascular risk related to Lp(a). One is to incorporate variants of the *LPA* region into CAD PRS. A study shows that the *LPA* region contributed 7.2 % of CAD-PRS-mediated risk of CAD [42]. Another involves Lp(a)-specific PRS which focuses directly on predicting genetically elevated Lp(a) levels by integrating LPA SNPs, KIV-2 copy number variation, and structural variants [40, 43, 44]. These models improve the prediction of elevated Lp(a) and CVD risk and identify genetically high-risk individuals, highlighting the potential of clinical implementation.

### Atrial fibrillation and heart failure

Atrial fibrillation (AF) and heart failure (HF) frequently coexist, share common risk factors and show a genetic correlation [45]. The development of HF following AF is particularly common and a clinically significant complication, emphasizing the need for improved risk stratification tools capable of identifying high-risk individuals early. Large-scale GWAS have identified hundreds of loci associated with AF, enabling the construction of PRS for its prediction [3].

Given the established bidirectional, “fire and fury” relationship between AF and HF [45], the question is whether the performance of one disorder’s PRS can predict the risk of the other. An exploratory study showed that the AF-PRS had the strongest association with HF (HR = 1.47) compared to other PRS models for CAD, blood pressure, and diabetes mellitus [46]. Further, a study utilizing the UK Biobank demonstrated that HF-PRS was strongly associated with incident HF in patients with AF, an effect particularly pronounced in younger patients (age < 60), where a moderate-to-high HF-PRS conferred a hazard ratio of 2.14 compared to a low PRS. Incorporating HF-PRS with clinical risk factors would reclassify 30 % to the high HF risk group in young AF, thereby underscoring the clinical value of genetic information for integrated AF management [47].

These observations indicate that PRS may provide incremental prognostic value for identifying AF patients who are at increased risk of developing HF. These patients should undergo early rhythm control including ablation therapy as preventive measures. In addition, PRS allows for better identification of HF patients with an increased risk of AF in whom regular screening for AF should be performed. Both scenarios showed greater effects of PRS in young people. Future studies will need to integrate genomic risk with ECG data, imaging data and clinical risk factors to further improve risk prediction and preventive strategies in AF and HF.

### Cardiomyopathies

PRS are increasingly being explored in cardiomyopathies, which were historically considered as monogenic diseases. Dilated cardiomyopathy (DCM) affects approximately 1 in 250–400 individuals and remains a major cause of HF and life-threatening arrhythmias despite improved diagnostics and therapies. Rare pathogenic variants in genes such as *TTN*, *DSP*, *MYH7*, *BAG3*, *TNNT2*, and *TPM1* account for around 25 % of cases, but many patients do not carry an identifiable disease-causing variant [48].

Recent GWAS have identified numerous common variants associated with cardiac structure, function, and DCM risk [49 – 51]. The respective polygenic variance can modify cardiac phenotypes among carriers of pathogenic variants (e.g., *TTN* truncating variants) and influence the penetrance of rare disease-causing variants[51]. Whether the addition of PRS to clinical risk factors may also improve the prediction of life-threatening arrhythmias in patients with DCM – potentially resulting in earlier recommendations for an implantable cardioverter-defibrillator (ICD), similar to patients with so-called “high-risk” mutations (e.g., *DSP*, *LMNA*, *FLNC*, and others) [8] – remains currently unknown. Moreover, DCM-PRS can predict the risk of incident DCM in individuals not carrying known mutations. Taken together, PRS in DCM not only enable the assessment of genetically determined risk in genotype-negative patients but also act as an important genetic modifier among patients with rare variants, influencing disease penetrance and underscoring its clinical potential for risk stratification and individualized clinical decision-making.

Hypertrophic cardiomyopathy (HCM) was long considered a purely monogenic disorder of the cardiac sarcomere, but with more than 50 % genotype negative cases and a considerable phenotypic heterogeneity, a broader genetic architecture has been proposed. Recent large GWAS-based studies demonstrated that common variants and their summation as PRS significantly influence HCM risk in the general population and strongly modify disease penetrance among carriers of rare pathogenic variants. Thereby, individuals in the highest PRS quintile showed up to a 10-fold higher disease penetrance compared with those in the lowest quintile. However, risk and severity of phenotypes remained significantly lower in high PRS individuals than in carriers of pathogenic sarcomeric variants [52].

Regarding risk stratification for ventricular arrhythmias and death in patients diagnosed with HCM, a high PRS was associated with higher mortality, an observation that was replicated in sarcomere positive patients [53]. Yet, prospective longitudinal studies analysing arrhythmias and sudden cardiac death in HCM patients with high versus low PRS are lacking.

**Figure 3:**
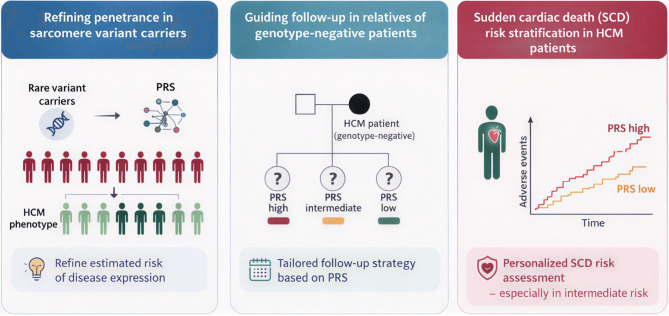
Clinical scenarios in which PRS can add to disease susceptibility and risk prediction in Hypertrophic cardiomyopathy (HCM).

From a clinical perspective, PRS in HCM may be particularly useful in three scenarios (Figure 3). First, it may help refine penetrance estimates in carriers of sarcomere variants. Second, it may guide follow-up strategies in relatives of genotype-negative patients. Third, PRS may provide an additional tool for sudden cardiac death risk stratification in patients with established HCM. In particular, patients with intermediate risk may benefit from a personalized risk assessment that incorporates PRS.

#### Hypertension

Hypertension is a complex, multifactorial trait with a substantial genetic component. Family and twin studies estimate the heritability (*h^2^*) of blood pressure to be in the range of 0.4–0.6 [54], indicating a significant genetic contribution to inter-individual variation.

PRS for blood pressure (BP) and hypertension have evolved dramatically from a few risk loci to clinically informative risk predictors. Nowadays, compared to the bottom decile, the top idecile of a BP-PRS revealed about 17 mmHg higher SBP, and a 7-fold higher odds of hypertension. Adding PRS to hypertension-prediction models, including age, gender, and body mass index, improved prediction performance to AUC 0.83 [4]. A multi-ethnic BP-PRS showed the strongest association with hypertension by OR = 2.10 and AUC = 0.76, indicating that integration of diverse populations may further improve risk prediction [55].

The clinical application of BP-PRS is being explored in the PRISM-BP (Polygenic Risk Score Implementation and Stratification for Managing Blood Pressure) trial (ClinicalTrial.gov ID NCT06962488), which tests whether disclosing the BP-PRS information with genetic counselling can make changes in blood pressure and motivate a healthy lifestyle in 300 young participants (18–55 years old) with hypertension and poor cardiovascular health.

Obviously, from a clinical point of view, genetic prediction of arterial hypertension can be easily overruled by direct measurements. Therefore, it would be more interesting to determine whether PRS can be used to guide treatments in terms of personalized antihypertensive therapy. These pharmacogenomic data were analysed in the GenHAT (Genetics of Hypertension Associated Treatment) [56]. In this BP-PRS study among 3745 Black individuals with hypertension, those in the lowest genetic risk quintile had a significantly greater BP reduction (approximately 3.5 mmHg) with the diuretic chlorthalidone compared to those with median PRS risk, while individuals in the highest PRS quintile had 67 % higher odds of developing apparent treatment-resistant hypertension. These findings suggest that PRS may have potential utility in stratifying treatment response and identifying individuals at higher risk of resistant hypertension; however, further studies in diverse populations are required to determine its clinical applicability.

#### Aortic aneurysm and valvular heart disease

Thoracic aortic dilation is a major risk factor for aortic dissection, a mostly catastrophic, sudden onset event. ESC guidelines define dilation as a diameter ≥4.0 cm of the ascending aortic [57]. Currently, screening for thoracic aortic dilatation is only recommended in first-degree relatives of someone with thoracic aortic aneurysm or aortic dissection and universal imaging is impractical. Clinical risk models have therefore been developed to estimate ascending aortic diameter and identify individuals who may benefit from early imaging. These models explain up to 30 % of the variance in aortic size achieving moderate predictive performance. Given that ascending aortic diameter is highly heritable, PRS can now provide additional value in risk estimation. Importantly, in a recent study the polygenic information alone explained more of the variance in thoracic aortic diameter compared to clinical factors alone: 39.5 % vs 29.3 % [58]. Integration of PRS with clinical risk factors accounted for 35 – 42 % of the variance in ascending aortic diameter. Besides providing enhanced identification of ascending aortic dilation, integration of the PRS was strongly associated with adverse events including thoracic aortic dissection beyond clinical risk factors alone [58].

**Figure 4:**
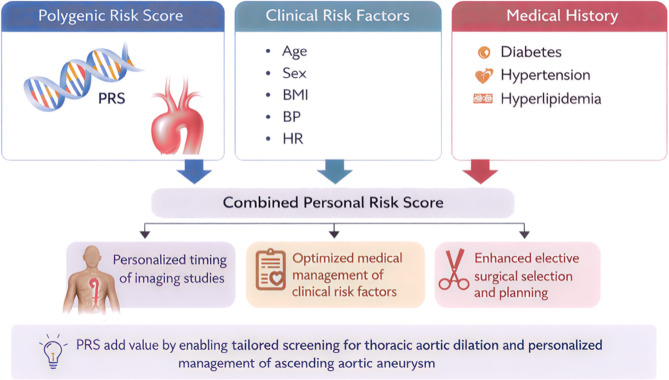
**Combined risk estimation using PRS and clinical risk factors for ascending aortic dilatation and management of ascending aortic aneurysm** (modified from Pirrucello et al EHJ 2024). BP, blood pressure; HR, heart rate.

Therefore, in future clinical practice, aortic aneurysm PRS may support individualized decisions regarding imaging of the aorta (screening and follow-up) as well as optimization of medical therapy and lifestyle, including sports in patients with aortic aneurysms, and most importantly the timing of surgical intervention through improved risk-benefit assessment (Figure 4)

Another cause of aortic aneurysm may be a concomitant bicuspid aortic valve (BAV) in which the genetic basis was thought to arise from rare familial forms with monogenic inheritance and incomplete penetrance. However, accumulating evidence from GWAS indicates that common genetic variation also contributes to the development of these conditions, suggesting a broader polygenic genetic architecture. In BAV, the largest GWAS meta-analysis to date of nearly 10 000 cases identified 36 genetic loci, highlighting a strong polygenic contribution. Further, a calculated PRS was strongly associated with BAV risk (OR ≈ 2.1 per SD) [58]. Whether the risk of concomitant aortic aneurysm in BAV can be estimated via PRS is currently under investigation.

Similarly, in mitral valve prolapse (MVP), recent GWAS meta-analyses have identified numerous loci involved in connective tissue and developmental signalling pathways, enabling the development of a PRS [59]. Thereby, it would be of high clinical relevance whether PRS may help to identify those individuals with MVP who also carry a high risk for ventricular arrhythmias so-called “malignant mitral valve prolapse” syndrome. However, so far large-scale genetic analyses of MVP cases with and without arrhythmias are lacking

## Gaps of knowledge and future outlook

Polygenic risk scores hold great promise for transforming cardiovascular prevention and therapy by enabling earlier and more individualized risk assessment and therapy. Realizing this potential, however, will require moving beyond statistical prediction toward clinically meaningful implementation. It will be crucial to integrate genomic data with available clinical and lifestyle-based risk factors and establish clear pathways for how PRS information can guide clinical decision-making. In parallel, clinicians must be educated in PRS interpretation to understand their strengths and limitations to translate results into actionable guidance for patients. Further, continued validation of estimates in prospective clinical studies will be essential, alongside efforts to ensure robust performance across diverse populations. If these challenges can be addressed, PRS will evolve from a research tool into a routine component of cardiovascular risk assessment.
